# The Effect of HCV on Methadone Dose During Pregnancy

**DOI:** 10.1111/jvh.70060

**Published:** 2025-08-04

**Authors:** Sarah Boudova, Neel S. Iyer, Danielle M. Tholey, Jonathan M. Fenkel, Rupsa C. Boelig

**Affiliations:** ^1^ Division of Maternal‐Fetal Medicine, Department of Obstetrics and Gynecology, Sidney Kimmel Medical College Thomas Jefferson University Philadelphia Pennsylvania USA; ^2^ Division of Gastroenterology and Hepatology, Sidney Kimmel Medical College Thomas Jefferson University Philadelphia USA

**Keywords:** hepatitis C virus, methadone, opioid‐use disorder, pregnancy

## Abstract

Pregnancy is a time of high patient motivation to initiate treatment for opioid use disorder (OUD). Hepatic drug metabolism can be altered by pregnancy and hepatitis C virus (HCV) infection. We aimed to examine the impact of HCV during pregnancy on methadone dosing. Retrospective chart review of all pregnant patients with OUD admitted for initiation of methadone from 1/2020–6/2022. Associations were examined using Student's T‐tests, chi‐squared tests, Fisher's exact tests and univariate and multivariate linear regression. We identified 191 pregnancies initiated on methadone, of which 188 were screened for HCV. 111 (59.0%) were HCV Antibody (Ab)+, of whom 108 were tested for HCV RNA and 66 (61.1%) were HCV RNA+. The median viral load was 498,500 IU/mL (range 19–46,000,000 IU/mL). Fibrosis‐4 (Fib4) score, an estimate of liver fibrosis, was available for 97 pregnancies. The average Fib4 score was 0.36 (SD 0.69), and only five individuals had Fib4 scores > 1.45. White race (*p* < 0.001) and injection drug use (*p* < 0.001) were associated with being HCV RNA+. HCV RNA+ individuals had higher Fib4 scores (*p* = 0.022). We found no association between being HCV RNA+ and stable methadone dose achieved during hospitalisation (*p* = 0.105) in univariate analysis or a multivariate linear regression model (*p* = 0.567). There was no correlation between viral load or Fib4 score and stable methadone dose. No patient had a Fib4 score > 3.25. Our data suggest that HCV‐specific alterations are unnecessary for methadone dosing in pregnancy and that fibrotic liver damage is rare in this population. However, further research is warranted for the subset of pregnant patients with advanced fibrosis.

## 1 Introduction

Opioid use disorder (OUD) has become increasingly common in pregnant patients, nearly quadrupling since 1999 [1]. Approximately 8.2 per 1000 pregnancies are estimated to be complicated by OUD [2], with significant health consequences. It is associated with preterm birth, stillbirth, neonatal abstinence syndrome and long‐term neurocognitive impairment [3, 4]. It is also a leading cause of maternal mortality [5, 6, 7, 8]. Individuals with OUD have a six‐fold higher incidence of all‐cause postpartum death [9].

Thus, pregnancy is a critical time to intervene to minimise adverse maternal and foetal health outcomes [10]. Moreover, pregnancy is a period of high patient motivation and engagement [11] when patients who may not otherwise have healthcare are insured, potentially mitigating barriers to treatment.

Methadone is commonly used to treat OUD in pregnancy [12, 13]. Methadone is primarily metabolised in the liver through cytochrome P450, and predominantly by cytochrome P450 2B6 (CYP2B6) [14]. Pregnancy alters the metabolism of methadone due to increased activity of hepatic enzymes, as well as the increased volume of distribution [15, 16]. Thus, pregnant patients often require higher doses of methadone [17].

With the rise in OUD, there has been a concomitant increase in hepatitis C virus (HCV) infection [18, 19]. The prevalence of HCV‐infected pregnancies in the US increased 16‐fold between 1998 and 2018 [20]. Among patients with OUD, 216.9 per 1000 pregnancies are complicated by HCV infection [18]. The liver is the primary target of HCV infection, which causes acute hepatitis, chronic inflammation, fibrosis, cirrhosis and increases the risk for hepatocellular carcinoma [21]. It is thought that HCV infection impacts hepatic drug metabolism and thus may reduce methadone dose requirements [22, 23]. Notably, Wu et al. showed a significant reduction in methadone metabolism in HCV antibody (Ab)+ patients [24]. Unfortunately, the authors of this study did not capture HCV RNA status, which is a more accurate metric of current HCV infection. Other literature has suggested altered methadone metabolism in the setting of HCV may be due to decreased CYP2B6 enzyme activity [24, 25, 26]. However, none of these studies examined the impact of HCV on methadone metabolism, and consequently, methadone dose, in pregnancy. Taking advantage of a well‐characterised cohort of pregnant patients initiated on methadone during pregnancy, we aimed to determine the association between being HCV RNA+ and stable methadone dose achieved during hospitalisation during pregnancy and stable methadone dose at delivery.

## 2 Methods

### 2.1 Research Design and Study Sample

This is a secondary analysis of a retrospective cohort study of pregnant patients admitted for methadone initiation or titration at Thomas Jefferson University Hospital (Philadelphia, PA) between January 2020 and June 2022 [17]. Patients were initiated or up‐titrated on methadone based on their clinical opioid withdrawal score (COWS) per institutional protocol, as previously described [17]. The patient was considered to be at a stable methadone dose if they did not require any additional 10 mg doses for 12–24 h based on the institutional protocol. Of note, patients who achieved a stable methadone dose during hospitalisation may have required additional outpatient methadone titration due to the physiologic changes of pregnancy, resulting in a different methadone dose at the time of delivery. This study was approved by the Thomas Jefferson University Hospital Institutional Review Board. Data were abstracted from the electronic medical record on age, race, ethnicity, gravidity, parity, gestational age at the time of admission, BMI, self‐reported substance use, history of injection drug use (IDU), tobacco use, alcohol use and history of prior substance use disorder treatment. Additional laboratory and clinical data included: presence of human immunodeficiency virus (HIV) infection, hepatitis B infection, HCV antibody screening, HCV viral load, fibrosis 4 (Fib4) score (an index of cirrhosis), aspartate transaminase (AST), alanine transaminase (ALT), length of admission, time to stable methadone dose achieved during hospitalisation, stable methadone dose achieved during hospitalisation, stable methadone dose at the time of delivery, elopement prior to achievement of stable methadone dose, number of methadone‐related readmissions, HCV genotype and hepatic ultrasound results. Patients who had a positive antibody test were considered to have screened positive for HCV and designated HCV Ab+. Patients with detectable viral load were designated HCV RNA+, and were considered to be infected with HCV. Patients who were HCV Ab−, and thus did not have an HCV viral load performed, were assumed to be HCV RNA−. Patients without antibody screening, or those who were antibody positive but not tested for HCV RNA, were excluded from analysis. Fib4 score was calculated to assess liver damage as previously described [27].

### 2.2 Outcomes

The primary outcome was the association between being HCV RNA+ and stable methadone dose achieved during hospitalisation. Secondary outcomes included the association between viral load and stable methadone dose achieved during hospitalisation, and the association between Fib4 score and stable methadone dose achieved during hospitalisation.

### 2.3 Statistical Methods

Descriptive statistics, including mean, standard deviation, median, range and frequency, were calculated. Student's t‐tests were used to compare continuous variables. Chi‐squared tests and Fisher's exact tests were used to compare categorical data. *p*‐values were two‐sided, and *p* < 0.05 was used as the threshold for statistical significance. Univariate logistic regression of association with stable methadone dose achieved during hospitalisation was performed, and any variables with *p* < 0.1 were included in a multivariate linear regression model of association between being HCV RNA+ and stable methadone dose achieved during hospitalisation. All statistical analyses were conducted using Stata17 software (StataCorp LLC, College Station, TX). Graphs were created using GraphPad Prism 9 for Windows (GraphPad Software, San Diego, CA).

## 3 Results

**FIGURE 1:**
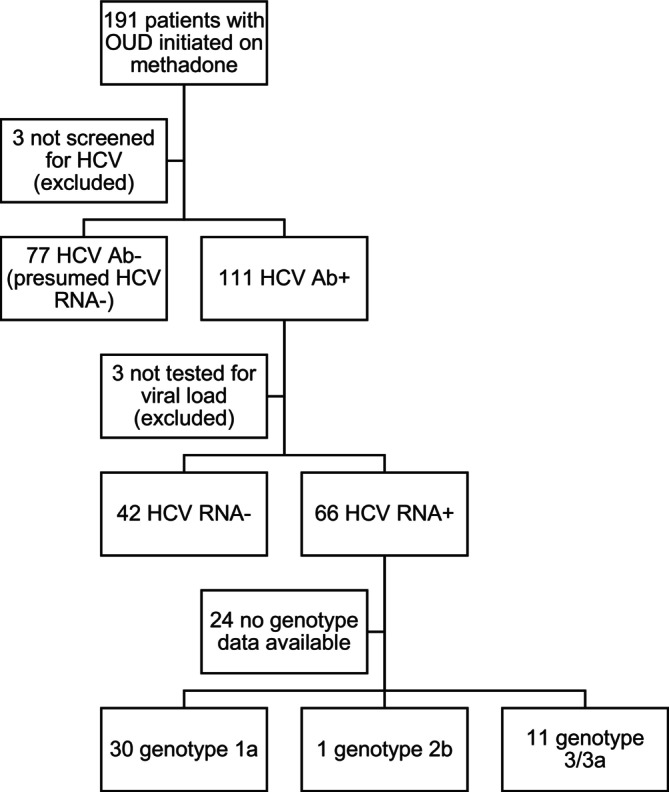
HCV testing flow diagram. Study flow diagram of HCV testing. Abbreviations: Ab, antibody; HCV, hepatitis C virus; OUD, opioid use disorder; RNA, ribonucleic acid.

We identified 191 pregnancies in patients with OUD initiated on methadone. Among these, 188 (98.4%) were screened for HCV: 77 (41.0%) were HCV Ab− and 111 (59.0%) were HCV Ab+ (Figure 1). Among those who were HCV Ab+, 108 had a viral load collected (97.29%): 42 (38.9%) were HCV RNA− and 66 (61.1%) were HCV RNA+. Genotype data were available for 42 (63.6%) of these infections. Type 1a was most common (30, 71.4%), followed by type 3/3a (11, 26.2%) and type 2b (1, 2.4%).

We examined demographics based on whether or not the patient was HCV RNA+. White race (*p* = 0.001), heroin use (*p* = 0.018), fentanyl use (*p* < 0.001), IDU (*p* < 0.001), Fib4 score (*p* = 0.022), AST (*p* < 0.001) and ALT (*p* < 0.001) were significantly associated with being HCV RNA+ (Table 1). Oral opioid use was significantly associated with being HCV RNA‐ (*p* < 0.001). There was no significant difference in age, gravidity, parity, gestational age at time of admission, Hispanic ethnicity, BMI, tobacco use, alcohol use, history of prior substance use treatment, HIV infection or hepatitis B infection based on whether an individual was HCV RNA+.

**TABLE 1:** Demographic characteristics.

	HCV RNA+ (*n* = 66)	HCV RNA− (*n* = 119)	*p*
Age (mean, SD)	31.11 (4.60)	30.93 (4.88)	0.814
Gravidity (mean, SD)	3.61 (2.01)	3.77 (2.45)	0.637
Parity (mean, SD)	1.45 (1.47)	1.48 (1.47)	0.911
Gestational age in weeks (mean, SD)	19.00 (9.85)	18.62 (9.66)	0.801
Race (*n*, %)			
White	55 (83.3%)	83 (69.75%)	
Black	3 (4.55%)	28 (23.53%)	
Asian	0	1 (0.84%)	**0.001** ^a^
Other	8 (12.12%)	7 (5.88%)	
Hispanic ethnicity (*n*, %)	9 (13.63%)	11 (9.24%)	0.357
BMI (mean, SD)^b^	24.98 (5.70)	26.45 (6.21)	0.094
Self‐reported substance use (*n*, %)			
Heroin Fentanyl Oral opioids Marijuana Benzodiazepines Cocaine Amphetamines PCP Ketamine Xylazine Other	61 (92.42%) 65 (98.48%) 0 6 (4.80%) 14 (21.21%) 26 (39.39%) 9 (13.63%) 1 (1.52%) 1 (1.52%) 0 1 (1.52%)	94 (78.99%) 92 (77.31%) 19 (15.97%) 23 (19.33%) 26 (21.85%) 31 (26.05%) 12 (10.08%) 3 (2.52%) 0 1 (0.84%) 3 (2.52%)	**0.018** **< 0.001** **< 0.001** ^a^ 0.067 0.920 0.060 0.466 0.652 0.357^a^ 1.000^a^ 0.652
History of injection drug use (*n*, %)	65 (98.48%)	61 (51.26%)	**< 0.001**
Tobacco use in pregnancy (*n*, %)^c^	55 (85.94%)	94 (81.03%)	0.404
Alcohol use in pregnancy (*n*, %)^d^	6 (9.52%)	5 (4.46%)	0.186
History of prior substance use treatment (*n*, %)	45 (68.18%)	84 (70.59%)	0.733
HIV‐infected (*n*, %)^e^	2 (3.03%)	1 (0.85%)	0.262
Hepatitis B infected (*n*, %)^f^	1 (1.59%)	1 (0.85%)	0.650
Fib4 score (mean, SD)^g^	0.75 (0.05)	0.58 (0.23)	**0.022**
AST (mean, SD)^g^	41.10 (43.20)	15.80 (6.71)	**< 0.001**
ALT (mean, SD)^h^	49.44 (60.56)	13.22 (6.92)	**< 0.001**
Platelet count (mean, SD)^i^	273.30 (82.05)	266.54 (71.29)	0.666

*Note:* T‐test and Chi‐squared unless otherwise specified. *p* values < 0.05 were bolded.

Abbreviations: BMI, body mass index; HCV, hepatitis C virus; HIV, human immunodeficiency virus; PCP, phencyclidine; RNA, ribonucleic acid; SD, standard deviation.

^a^ Fisher's exact test.

^b^ Data available for 65 HCV RNA+ individuals.

^c^ Data available for 64 HCV RNA+ individuals and 116 HCV RNA− individuals.

^d^ Data available for 63 HCV RNA+ individuals and 112 HCV RNA− individuals.

^e^ Data available for 118 HCV RNA− individuals.

^f^ Data available for 63 HCV RNA+ individuals and 118 HCV RNA− individuals.

^g^ Data available for 62 HCV RNA+ individuals and 35 HCV RNA− individuals.

^h^ Data available for 64 HCV RNA+ individuals and 37 HCV RNA− individuals.

^i^ Data available for 64 HCV RNA+ individuals and 41 HCV RNA− individuals.

For our primary outcome, we examined stable methadone dose on discharge from hospital based on whether an individual was HCV RNA+. HCV RNA+ and HCV RNA‐ patients had no difference in mean stable methadone dose achieved during hospitalisation (137.31 +/− 96.62 mg vs. 114.58 +/− 65.15 mg, *p* = 0.105) (Table 2). Regarding secondary outcomes, HCV RNA+ patients had a significantly longer mean hospital admission length than HCV RNA− patients (7.24 +/− 5.90 days vs. 5.67 +/− 3.95 days, *p* = 0.032) (Table 2). Otherwise, there was also no difference in time to achieving stable dose, elopement from the hospital prior to achieving stable dose, number of methadone‐related readmissions or methadone dose at the time of delivery (Table 2).

**TABLE 2:** Methadone titration outcomes.

	HCV RNA+ (*n* = 66)	HCV RNA− (*n* = 119)	*p*
Admission length in days (mean, SD)	7.24 (5.90)	5.67 (3.95)	**0.032**
Time to stable dose in days (mean, SD)^a^	6.23 (4.20)	5.01 (2.97)	0.051
Stable dose in mg (mean, SD)^a^	137.31 (96.62)	114.58 (65.15)	0.105
Elopement from the hospital prior to stable dose (*n*, %)	14 (21.21%)	35 (29.41%)	0.226
Methadone‐related readmissions (*n*, %)^b^			
0 1 2 3 ≥ 4	13 (34,21%) 12 (31.58%) 7 (18.42%) 2 (5.26%) 4 (10.53%)	39 (43.82%) 22 (24.72%) 8 (8.99%) 5 (5.62%) 15 (16.85%)	0.435
Methadone dose at delivery (mean, SD)^c^	135.38 (103.74)	144.14 (106.60)	0.698

*Note:* *p* values < 0.05 were bolded.

Abbreviations: HCV, hepatitis C virus; RNA, ribonucleic acid; SD, standard deviation.

^a^ Among patients who did not elope from the hospital (*n* = 52 HCV RNA+, *N* = 83 HCV RNA−).

^b^ Among patients who delivered at our institution (*N* = 38 HCV RNA+, *N* = 89 HCV RNA−).

^c^ Among patients who were on methadone at delivery (*N* = 32 HCV RNA+, *N* = 71 HCV RNA−).

We next performed a univariate analysis of factors associated with stable methadone dose achieved during hospitalisation. We found that age was positively associated with increased methadone dose (coefficient 3.34, 95% CI 0.46–0.621, *p* = 0.023) and that Black race (coefficient −40.12, 95% CI −75.46 to −4.77, *p* = 0.026), oral opioid use (coefficient −49.86, 95% CI −90.38 to −9.34, *p* = 0.016) and PCP use (coefficient −98.30, 95% CI −187.62 to −8.96, *p* = 0.031) were associated with lower methadone dose (Table S1). There was no association between being HCV RNA+ and stable methadone dose achieved during hospitalisation (coefficient 22.73, 95% CI −4.81 to 50.27, *p* = 0.105) (Table S1). Similarly, there was no association between being HCV RNA+ and stable methadone dose achieved during hospitalisation in a multivariate logistic regression model accounting for age, race, IDU, oral opioid use, PCP use and AST (coefficient 14.20, 95% CI −35.03 to 63.44, *p* = 0.567) (Table 3).

**TABLE 3:** Multivariate linear regression of association with stable methadone dose achieved during hospitalisation.

	Coefficient	Standard error	*p*	95% CI
HCV RNA+	14.20	24.69	0.567	‐35.03 to 63.44
Age	0.96	2.30	0.677	−3.62 to 5.54
Race				
White Black Asian Other	Ref −0.96 ‐ 22.14	Ref 60.90 1 39.12	Ref 0.987 ‐ 0.573	Ref −122.41 to 120.49 ‐ −55.89 to 100.16
History of injection drug use	36.21	58.85	0.540	−81.16 to 153.59
Self‐reported substance use				
Oral opioids PCP	12.49 0	86.02—	0.885—	−159.07 to 184.05—
AST	−0.62	0.34	0.074	−1.31 to 0.06

Abbreviations: AST, aspartate aminotransferase; HCV, hepatitis C virus; PCP, phencyclidine; RNA, ribonucleic acid; SD, standard deviation.

**FIGURE 2:**
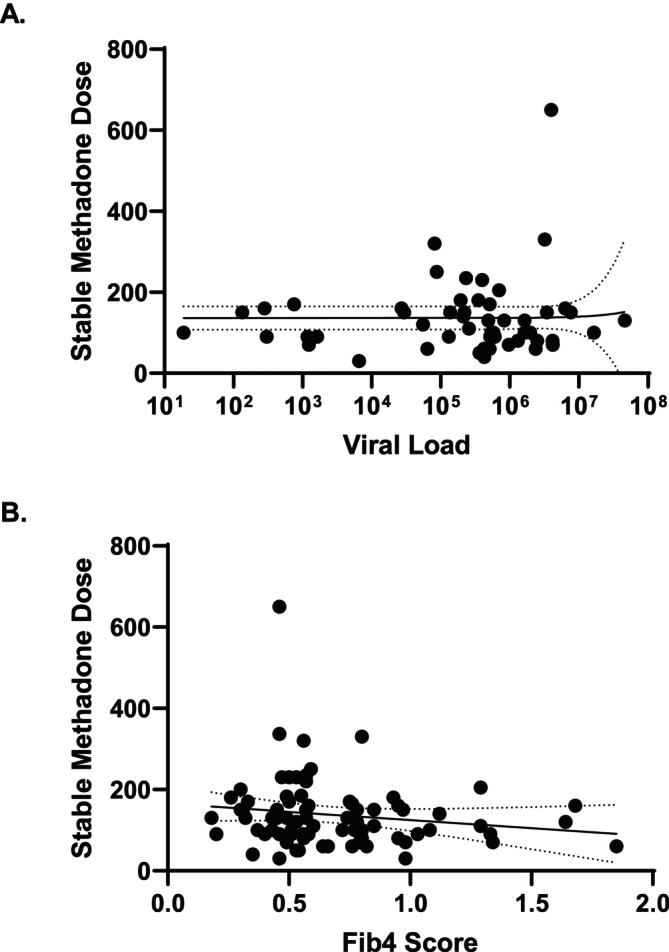
Association between HCV viral load and Fib4 score and stable methadone dose during hospital admission. A. Viral load in international units per millilitre is plotted against stable methadone dose in milligrams. *N* = 52. The X‐axis is log transformed. Linear regression of line of best fit with 95% CI. R‐squared is < 0.001. *p* value of line of best fit is 0.872. B. Fib4 score is plotted against stable methadone dose in milligrams. *N* = 78. Linear regression of line of best fit with 95% CI. R‐squared is 0.024. *p* value of line of best fit is 0.178.

We further plotted viral load versus stable methadone dose achieved during hospitalisation and found no association, with an R‐squared < 0.001 and *p* = 0.872 (Figure 2A). The median viral load was 498,500 IU/mL (range 19–46,000,000 IU/mL).

Finally, we examined the association between Fib4 score and stable methadone dose achieved during hospitalisation, and found no association, with an R‐squared of 0.024 and *p* = 0.178 (Figure 2B). The Fib4 score was available for 97 pregnancies. The average Fib4 score was 0.36 (SD 0.69, range 0.18–2.16), and only five individuals had Fib4 scores > 1.45. Right upper quadrant ultrasounds were performed on 63 patients. Four patients had cholelithiasis, two patients had gallbladder sludge, six patients had hepatic steatosis, two patients had benign liver masses (haemangiomas), one patient had coarsened hepatic echotexture and one patient had slightly increased attenuation of the portal triad. There was one patient with possible cirrhosis based on ultrasound imaging, showing a nodular liver surface.

## 4 Discussion

We found no association between being HCV RNA+ and the stable methadone dose achieved during hospitalisation in our pregnant population. This was shown in the univariate analysis as well as in a multivariate regression model that controlled for potential confounders identified in the univariate linear regression. We further saw no association between viral load and the methadone dose achieved during hospitalisation. Together, our data suggest that being HCV RNA+ does not appear to have a clinically significant impact on the methadone dose in pregnancy and adjustments to dosing based on HCV history are unnecessary.

Our data stand in contrast to work by Wu et al., who showed that HCV Ab+ patients had decreased methadone metabolism [24]. There are several potential explanations for this difference. Wu did not examine whether or not patients were HCV RNA+, but simply if they were HCV Ab+, which is suggestive that HCV may decrease metabolism of methadone, but without data on HCV RNA status, this cannot be certain. Alternatively, metabolic differences seen by Wu, while statistically significant, may not be enough to change clinical dosing, or the metabolic changes of pregnancy may negate the potential effect of HCV. It could also be due to patient population differences. Wu et al. studied a Han Chinese population in which 95% were HCV Ab+ and 23.58% were HIV positive. In contrast, only one of our patients identified as Asian, only three were HIV positive and 59.0% of our patients were HCV Ab+. It has been shown that HIV/HCV coinfected patients are more likely to have alterations in methadone dosing than those with HCV mono‐infection [28], potentially explaining our difference. However, we think that the most probable reason for this difference is the degree of hepatic damage. Wu noted significant elevation in AST and ALT in their HCV Ab+ population, while we saw very limited liver damage in our population. Differences in HCV genotype may play a role in this. Some genotypes are more likely to cause fibrosis. To this effect, Sutlović et al. found that the only major influence on methadone metabolism in HCV‐infected patients was the stage of liver damage [29].

While being HCV RNA+ was common in our population (69/185, 37.3%), few patients in our study had evidence of fibrotic liver damage. Most (90/95, 94.74%) had Fib4 scores less than 1.45, which has a high negative predictive value for advanced fibrosis [27]. No patients had Fib4 scores over 3.25, which has a high positive predictive value for advanced fibrosis. Only 6 patients had evidence of hepatic steatosis noted on ultrasound, and only one had ultrasound findings suggestive of cirrhosis. The lack of correlation between Fib4 score and stable methadone dose achieved during hospitalisation in our study may be the result of a type 2 error due to our limited sample of patients with advanced fibrosis. The lack of hepatic damage in our study is likely due to the young age of the pregnant population and consequent relatively recent infection without time for chronic inflammatory damage to occur. Additional studies focusing on a larger cohort of patients with advanced fibrosis or cirrhosis would be valuable in instructing methadone dosing in patients with advanced liver disease.

Interestingly, while we did not see a difference in the methadone dose or time to achieve a stable methadone dose, we did observe that HCV RNA+ patients had a significantly longer hospital course than HCV RNA− patients, staying almost 2 days longer on average. This was likely due to comorbid conditions that were also being treated during the hospital stay. HCV‐infected pregnant patients have been shown to score higher on the baseline Charlson‐Deyo Comorbidity Index than their HCV‐uninfected peers [20]. The exact nature of these comorbidities, and whether they can be reduced with HCV or OUD treatment, is an area for future research.

Our study has several limitations. With only 191 patients available in our data set, we may have been underpowered to detect a difference in stable methadone dose achieved during hospitalisation, especially in patients with advanced fibrosis or cirrhosis. In a post hoc power analysis, we had 40.1% power to detect a difference in stable methadone dose achieved during hospitalisation based on HCV status. Only five patients had Fib4 scores over 1.45, and no patient had a Fib4 score over 3.35; thus, these findings likely do not apply to patients with advanced fibrosis or cirrhosis. Indeed, outside of pregnancy, fibrotic liver damage has been shown to be the primary driver of changes in methadone dosing in HCV+ individuals [29]. However, the lack of liver disease in the pregnant population is an important finding as it presents an opportunity for treating HCV prior to the development of cirrhosis. Additionally, high levels of patient engagement with OUD treatment [11] make pregnant individuals an ideal population to target for HCV treatment. Bundling HCV and OUD treatment presents an opportunity to improve maternal, neonatal and population‐level health. HCV treatment could be coupled with paediatric or OUD therapy appointments to maximise completion of direct‐acting antiviral therapy, as has been successfully demonstrated in a study where treatment rates more than doubled [30].

A further limitation of this study is that while we were able to collect data on the dose of methadone received, we have no pharmacokinetic data on the plasma methadone or methadone metabolite levels to assess biochemical differences in methadone metabolism that would not be observed through COWS scoring. However, we believe that the dose of methadone received is the most clinically relevant parameter. We also have no data on patient cytochrome p450 genetic polymorphisms, which could influence methadone dose [29]. While we were able to collect data on Fib4 score and hepatic ultrasound, we do not have biopsy or elastography data, which would provide a more accurate diagnosis of liver fibrosis and cirrhosis. This study focused on HCV and methadone dosing; similar research should be done with buprenorphine, the other commonly used medication to manage OUD in pregnancy. Additionally, we grouped all HCV RNA− individuals together for our analysis, regardless of antibody status. There may be different risk factors and thus methadone requirements between individuals who had been exposed to HCV and those who have not, as well as those who spontaneously cleared infection versus those who were treated for HCV. With a larger study population, it would be worth examining if there are differences between these subgroups. Finally, this data is from a single, urban, tertiary centre, limiting its external validity.

Despite these limitations, there are many strengths to our study. As a referral centre for patients with opiate use disorder in pregnancy, with a significant percentage infected with HCV, we were able to report on an understudied question in a time when both HCV and opiate use in pregnancy continue to rise. Additionally, we examined HCV infection in a number of ways, looking at HCV RNA+ status, evaluating viral load itself and examining detailed markers of liver injury. We were also able to evaluate methadone dosing with multiple metrics, including stable discharge dose, time to achieve stable dose, number of methadone‐related readmissions and final dose at delivery. There was no difference in any of these metrics based on HCV status. Other strengths include our ability to control for multiple potential confounding variables using a multivariate model and the availability of data on hepatic health.

## 5 Conclusions

HCV infection is common in pregnant patients with OUD. We found no correlation between stable methadone dose achieved during hospitalisation and HCV infection or viral load, suggesting that HCV‐specific alterations are unnecessary for methadone dosing in pregnancy. We further observed minimal evidence of hepatic damage in these patients. However, additional studies evaluating whether methadone dosing remains stable in patients with advanced fibrosis and cirrhosis would be beneficial.

## Conflicts of Interest

J.M.F. receives research support to the institution from AbbVie and Gilead. R.C.B. receives research funding from Covis Pharma, Pharmacosmos, NICHD, March of Dimes, EW Thrasher Foundation, Children's Investment Fund Foundation and Bill and Melinda Gates Foundation. S.B. receives research funding from the EW Thrasher Foundation and Merck Investigator Initiated Studies Program.

## Supporting information


Appendix S1.

## Data Availability

The data that support the findings of this study are available from the corresponding author upon reasonable request.
