# Supplementary material for: The Effect of HCV on Methadone Dose During Pregnancy
Source: J Viral Hepat. 2025 Aug 4;32(9):e70060. doi: 10.1111/jvh.70060 (PMC12320570; doi:10.1111/jvh.70060)
Supplement: Supplementary file 1 — Appendix S1. [file JVH-32-0-s001.docx]

**Appendix**

Supplemental Table 1: Univariate linear regression of association with stable methadone dose

|  | Coefficient | Standard Error | p-value | 95%CI |
| --- | --- | --- | --- | --- |
| HCV RNA+ | 22.73 | 13.92 | 0.105 | -4.81 – 50.27 |
| Age | 3.34 | 1.46 | **0.023** | 0.46 – 6.21 |
| Gravidity | 0.47 | 2.85 | 0.868 | -5.15 – 6.10 |
| Parity | 0.002 | 0.004 | 0.631 | -0.007 – 0.011 |
| GA | -0.41 | 0.70 | 0.564 | -1.779 – 0.98 |
| Race  White  Black  Asian  Other | Ref  -40.12  -  5.55 | Ref  17.87  -  23.64 | Ref  **0.026**  -  0.815 | Ref  -75.46 - -4.77  -  -41.19 – 52.30 |
| Ethnicity | 26.37 | 21.41 | 0.220 | -15.97 – 68.71 |
| BMI | 1.21 | 1.15 | 0.296 | -1.07 – 3.48 |
| Self-reported substance use  Heroin  Fentanyl  Oral opioids  Marijuana  Benzodiazepines  Cocaine  Amphetamines  PCP  Ketamine  Xylazine  MDMA  Other | 25.26  27.48  -49.86  21.23  -8.85  11.16  -28.30  -98.30  -  87.81  -  -12.92 | 18.18  18.51  20.49  17.89  16.04  14.44  19.30  45.17  -  78.65  -  79.00 | 0.167  0.140  **0.016**  0.237  0.582  0.441  0.145  **0.031**  -  0.266  -  0.870 | -10.68 – 61.21  -9.11 – 64.08  -90.38 - -9.34  -14.14 – 56.61  -40.56 – 22.87  -17.38 – 39.71  -66.46 – 9.86  -187.62 - -8.96  -  -67.72 – 243.34  -  -169.15 – 143.31 |
| History of injection drug use | 24.43 | 14.41 | 0.092 | -4.07 – 52.93 |
| Tobacco use in pregnancy | 14.63 | 17.52 | 0.405 | -20.04 – 49.29 |
| Alcohol use in pregnancy | 3.53 | 29.42 | 0.905 | -54.69 – 61.76 |
| History of prior substance use treatment | 18.24 | 14.80 | 0.220 | -11.03 – 47.50 |
| HIV-infected | 26.69 | 78.86 | 0.736 | -129.27 – 182.65 |
| Hepatitis B-infected | 51.20 | 65.09 | 0.433 | -77.55 – 179.95 |
| Fib4 score | -40.06 | 29.48 | 0.178 | -98.77 – 18.66 |
| AST | -0.59 | 0.30 | 0.062 | -1.15-0.03 |
| ALT | -0.30 | 0.19 | 0.118 | -0.68 – 0.08 |

CI= confidence interval, HCV= hepatitis C virus, RNA= ribonucleic acid, BMI= body mass index, PCP= phencyclidine, MDMA= 3,4-methylenedioxymethamphetamine, HIV= human immunodeficiency virus, Fib4 = Fibrosis-4 (index of cirrhosis), AST= aspartate aminotransferase, ALT= alanine aminotransferase
